# Hydrophobic monomer systems for dental composites: development and physicochemical evaluation of UDMA/IBOMA formulations

**DOI:** 10.1186/s12903-026-08088-x

**Published:** 2026-03-14

**Authors:** Shaiva Thakar, Marc Hayashi, Zaher Jabbour, Reuben Kim, Shahed Al Khalifah, Deukwon Jo, Mijoo Kim

**Affiliations:** 1 UCLA Biomaterials and Device Testing Laboratory, UCLA School of Dentistry, Los Angeles, CA 90095 USA; 2 Section of Restorative Dentistry, UCLA School of Dentistry, 10833 Le Conte Ave, Los Angeles, CA 90095 USA

**Keywords:** Dental resin composite, UDMA, IBOMA, Bis-GMA, TEGDMA, Hydrophobicity, Water sorption, Contact angle, Restorative dentistry

## Abstract

**Background:**

The objective of this study was to evaluate UDMA/IBOMA formulations at varying ratios as hydrophobic alternatives to conventional Bis-GMA/TEGDMA dental resin composites, assessing hydrophobic properties, polymerization efficiency, mechanical performance, water sorption behavior, and color stability.

**Methods:**

A total of 120 disc specimens (15 mm × 1 mm) were prepared in four groups (*n* = 40): Group 1 (control) containing Bis-GMA: TEGDMA (70:30 wt%), and Groups 2–4 containing UDMA: IBOMA at 50:50, 60:40, and 70:30 wt%, respectively. The photoinitiators of 0.5 wt% Camphorquinone and 0.5 wt% Ethyl 4-dimethylaminobenzoate were added to each group. Specimens were evaluated for contact angle, surface free energy, degree of conversion (FTIR), Vickers microhardness, water sorption, and color stability (ΔE after 28-day immersion in coffee and turmeric). Data were analyzed using one-way and two-way ANOVA with Tukey’s HSD post-hoc test (α = 0.05).

**Results:**

All UDMA/IBOMA formulations exhibited significantly higher water contact angles (> 90°) and lower surface free energy than the control (*p* < 0.05). The degree of conversion showed no significant differences. Microhardness was comparable in Groups 2 and 3 but reduced in Group 4 (*p* < 0.05). Water sorption decreased progressively with increasing IBOMA content, with Group 4 demonstrating 71% reduction versus control (*p* < 0.05). UDMA/IBOMA formulations showed higher apparent staining for both solutions, primarily due to lighter baseline colors.

**Conclusions:**

The 70:30 UDMA/IBOMA formulation demonstrated superior water uptake resistance, representing significant advancement toward durable, BPA-free restorative materials with greatest potential for long-term clinical performance.

**Supplementary Information:**

The online version contains supplementary material available at 10.1186/s12903-026-08088-x.

## Background

Restorative dentistry plays a fundamental role in oral healthcare, encompassing the prevention, diagnosis, and treatment of pathological conditions affecting the teeth to restore their function and esthetics [1]. The global burden of dental disease underscores this need; the World Health Organization (WHO) reports that untreated dental caries alone affects approximately 2.5 billion people worldwide [2]. As the primary therapeutic intervention for arresting caries progression and replacing failing restorations, it is estimated that over 500 million direct dental restorations are performed annually worldwide [3]. Yet the clinical role of resin-based composites in contemporary dentistry extends well beyond caries management, encompassing an increasingly diverse range of indications. This expanding scope of application imposes complex mechanical and optical requirements that must be met to ensure clinical success across varied restorative contexts.

It is important to emphasize that dental resin composites are biphasic materials composed of an organic resin matrix and inorganic filler particles. While filler particles significantly influence mechanical properties such as wear resistance and compressive strength, the physicochemical stability and hydrolytic behavior of the composite are predominantly governed by the resin matrix. The polymer network constitutes the phase most susceptible to water absorption, which leads to plasticization of the matrix and potential degradation of the silane coupling agent at the filler-resin interface [4]. Consequently, optimizing the resin matrix chemistry to enhance hydrophobicity is the most direct strategy for improving the long-term hydrolytic stability and color longevity of the restoration, justifying the focus of this study on the monomer system.

While one of the widely used primary monomers, Bis-GMA, provides excellent mechanical stiffness, it possesses two significant limitations. First, it exhibits extremely high viscosity (≈ 1200 Pa·s), necessitating the use of low-viscosity diluents like triethylene glycol dimethacrylate (TEGDMA). High concentrations of TEGDMA, however, are associated with increased polymerization shrinkage and water sorption, which can compromise the clinical longevity of the restoration [4]. Second, Bis-GMA is derived from Bisphenol A (BPA), a known endocrine disruptor. The potential release of BPA or its derivatives via enzymatic degradation has raised biocompatibility concerns, prompting a surge in demand for “Bis-GMA-free” restorative materials [5].

In the search for alternatives, Ethoxylated Bisphenol-A Dimethacrylate (Bis-EMA) and Urethane Dimethacrylate (UDMA) have emerged as the primary candidates. Bis-EMA was developed to reduce viscosity by eliminating the hydroxyl (-OH) groups found in Bis-GMA, thereby removing the strong intermolecular hydrogen bonding that causes Bis-GMA’s high viscosity [6]. While this successfully facilitates handling and reduces water sorption, the lack of hydrogen bonding often results in polymers with lower flexural modulus compared to Bis-GMA based systems [7]. Because Bis-EMA-based formulations are widely represented in the literature on BPA-free/Bis-GMA-free restorative monomers, they provide important background context for positioning newer matrix designs. However, the present study was not designed as a direct experimental comparison with a Bis-EMA system; therefore, Bis-EMA is discussed here only to situate the UDMA/IBOMA approach within the broader landscape of alternative resin-matrix chemistries.

Conversely, UDMA offers a unique balance of properties. Introduced by Foster and Walker (1974) [8], UDMA possesses a flexible aliphatic backbone that lowers viscosity significantly (23 Pa·s) compared to Bis-GMA. Unlike Bis-EMA, UDMA retains the ability to form hydrogen bonds through its urethane linkages (-NH-), which imparts excellent toughness and a high degree of conversion [9]. Furthermore, UDMA is synthesized without a Bisphenol-A core, making it the most promising candidate for biocompatible, BPA-free formulations [10].

Despite its advantages, UDMA typically requires a co-monomer to fine-tune viscosity. The conventional diluent, TEGDMA, presents significant drawbacks. Structurally, TEGDMA is a low-molecular-weight, flexible linear molecule. This flexibility allows for primary intramolecular cyclization, a process where the molecule reacts with itself rather than crosslinking with the polymer network. This phenomenon consumes double bonds without contributing to network strength, leading to high polymerization shrinkage and heterogeneous network formation [11]. Furthermore, the ether linkages (-O-) in the TEGDMA backbone act as hydrogen bond acceptors, rendering the resin hydrophilic and prone to water sorption and hydrolytic degradation [12].

To overcome these drawbacks, we propose the use of Isobornyl Methacrylate (IBOMA) as a superior alternative reactive diluent. IBOMA is a monofunctional monomer distinguished by its bulky, hydrophobic bicyclic bornyl side group. Unlike linear diluents that reduce viscosity at the cost of polymer rigidity, IBOMA presents a unique structural advantage: its bulky side group prevents dense chain packing, effectively lowering the resin viscosity, while its rigid bicyclic structure maintains a high glass transition temperature (Tg ≈ 110 °C) via steric hindrance [9]. This allows for the formulation of low-viscosity composites that do not suffer from the mechanical “softening” typically associated with monofunctional diluents. Furthermore, as a high-molecular-weight monomer with only one polymerizable double bond, IBOMA reduces the overall concentration of double bonds per unit volume, theoretically lowering volumetric shrinkage compared to dimethacrylates like TEGDMA [13]. Finally, the hydrocarbon-rich isobornyl moiety renders the molecule highly hydrophobic, contrasting sharply with the hydrophilic ether linkages of TEGDMA, thereby offering superior resistance to water sorption and hydrolytic degradation [14].

Therefore, this study examined how different concentrations of UDMA and IBOMA influence key physicochemical properties, including contact angle and surface free energy, degree of conversion, Vickers microhardness, water sorption, and color stability, in comparison to a standard Bis-GMA/TEGDMA control material, with the goal of identifying formulations that offer superior hydrolytic resistance while maintaining acceptable mechanical and optical properties.

The null hypothesis states that varying the UDMA: IBOMA ratio would not affect (i) water contact angle and surface free energy, (ii) degree of conversion, (iii) Surface microhardness, (iv) water sorption, or (v) color stability (ΔE) relative to the Bis-GMA/TEGDMA control under the study conditions.

## Materials and methods

### Resin matrix formulation

The resin matrices were formulated using Bis-GMA, TEGDMA, UDMA, and IBOMA (Sigma-Aldrich Co., St. Louis, MO, USA). Group 1 served as the control, utilizing a 70:30 wt% ratio of Bis-GMA to TEGDMA. This composition was chosen to mimic the standard organic matrix of commercially available composites, optimizing the balance between viscosity and mechanical strength [15]. The experimental groups (Groups 2–4) were based on a formulation of UDMA and IBOMA as shown in Table 1.

**Table 1:** Composition of experimental resin matrices

Group	Resin matrix composition	Monomer Ratio (wt%)
Group 1 (Control)	Bis-GMA : TEGDMA	70:30
Group 2	UDMA : IBOMA	50:50
Group 3	UDMA : IBOMA	60:40
Group 4	UDMA : IBOMA	70:30

All groups contained 0.5 wt% camphorquinone and 0.5 wt% EDMAB as the photoinitiator system

A photoinitiator system comprising 0.5 wt% Camphorquinone (CQ) and 0.5 wt% Ethyl 4-dimethylaminobenzoate (EDMAB) was added to all mixtures. CQ served as the photosensitizer (absorption peak ~ 468 nm) and EDMAB as the co-initiator. This concentration was selected to optimize the degree of conversion while minimizing amine-induced discoloration [15].

Each monomer component was precisely weighed using a digital analytical balance scale (Model MR204, Mettler-Toledo GmbH, Greifensee, Switzerland) with an accuracy of ± 0.1 mg. The ingredients were then transferred into a mixing container shielded from ambient light. The base monomer blend was mixed first (Bis-GMA+TEGDMA or UDMA+IBOMA), followed by the addition of the photoinitiator components, and the mixture was homogenized using a magnetic stirrer hot plate (Fisher Scientific, Pittsburgh, PA, USA) at 45 °C temperature and 570 rpm for 60 min until a visually homogeneous resin blend was acquired. After mixing, the resin was allowed to stand for 120 min to permit gross bubbles to rise before specimen fabrication. No dedicated vacuum degassing/centrifugation step was performed.

### Specimen preparation

A total of 120 disc-shaped specimens (15 mm × 1 mm ± 0.05) were prepared using a metal mold, with each group consisting of 30 specimens. These dimensions were selected to satisfy the surface area requirements for all subsequent physical and optical property tests and comply with ISO 4049 [16].

The mold was placed on a glass slide, and the resin matrix was packed without entrapped bubbles. The top and bottom surface of the sample was covered with a Mylar strip to ensure a smooth, flat top surface and to prevent oxygen inhibition. Polymerization was performed on both top and bottom surfaces using a Valo Grand Deep Cure light-curing unit (1570 mW/cm²; Ultradent Products, Inc., South Jordan, UT, USA) for a total of 100 s, with 20 s exposures at five overlapping locations to ensure uniform curing across the entire diameter of the specimen. Following polymerization, all specimens were carefully inspected for the presence of internal or surface air voids under 5× magnification loupes, and any specimen exhibiting visible bubble incorporation was excluded from the study.

After polymerization and sample verification, samples were removed from the mold and stored in distilled water for 24 h at 37 °C to ensure complete polymerization and elution of residual monomers before testing.

### Contact angle measurement

The contact angle and surface free energy (SFE) were evaluated using five specimens from each group with disc dimensions (15 mm × 1 mm ± 0.05) prepared as described in the Specimen preparation section.

Measurements were performed using an optical tensiometer (Attension Theta Lite; Biolin Scientific, Gothenburg, Sweden) equipped with an automated sessile drop dispenser system. To determine the SFE, two probe liquids were used: distilled water (polar component) and di-iodomethane (dispersive/non-polar component) (Sigma-Aldrich Co., St. Louis, MO, USA).

A single drop of the test liquid was deposited on the surface of the sample. After the drop spread, a series of 30 images per second was recorded by a camera for 10 s. The software calculated the mean contact angle values. One reading was performed per sample for each liquid. The surface free energy was subsequently calculated using OneAttension software.

### Degree of Conversion (DC)

The degree of conversion (DC%) was determined using five specimens from each group prepared as described in the Specimen preparation section. Fourier-transform infrared (FTIR) spectra were obtained for the uncured monomer paste and for the top surface of the cured specimens after 24 h of storage in a dry, dark place.

To determine the degree of monomer conversion, an FTIR spectrometer (Thermo Fisher Scientific, Waltham, MA, USA) suitable for both solid and liquid sample analysis was used. The spectra were recorded within the 400–4000 cm⁻¹ range, at a resolution of 4 cm⁻¹ with 25 scans per sample. After 24 h of storage, each cured sample was carefully positioned onto the ATR crystal plate and secured with a clamp to ensure optimal contact.

The DC% was calculated based on the change in the absorption ratio of the aliphatic C = C band at 1637.645 cm⁻¹ relative to the internal reference aromatic C–C band at 1608.922 cm⁻¹. The following formula was used:

$$\begin{aligned}&\mathrm{D}\mathrm{C}\%=\\&[1-(\mathrm{A}_{1637}/\mathrm{A}_{1608})\mathrm{cured}/(\mathrm{A}_\mathrm{1637}/\mathrm{A}_{1608})\mathrm{uncured]}\\&\times100\end{aligned}$$

Where A represents the absorbance intensity of the respective bands.

### Surface microhardness

Surface microhardness was evaluated using five specimens from each group with a Micro Vickers microhardness tester (Phase II, Upper Saddle River, NJ, USA) equipped with a diamond pyramid indenter. A load of 1.96 N was applied with a dwell time of 15 s. Specimens were positioned on the tester platform, and to limit surface variations; three random indentations were made per surface.

Following indentation, samples were examined under a ×40 magnification lens. The diagonals of each pyramidal impression were measured using the device’s auto-measure software, with manual fine corrections applied as necessary. The Vickers Hardness Number (VHN) was automatically calculated by the device. For each surface, the mean of the three measurements was used as the representative microhardness value.

### Water sorption

A total of 20 samples were prepared for water sorption testing (five samples per group), using the specimen dimensions (15 mm × 1 mm ± 0.05) and curing protocol described in the Specimen preparation section.

Subsequently, the samples of each group were transferred and stored in a desiccator containing anhydrous self-indicating silica gel at 37 °C for 24 h. Following desiccation, the samples were weighed using a digital analytical balance with an accuracy of 0.1 mg; this initial dry mass was recorded as m₁.

After weighing, the dimensions of each sample were measured to calculate the volume (V). Two diameters perpendicular to each other were measured using an electronic digital caliper (0.01 mm accuracy) and averaged. The thickness was measured at the center and at four equally spaced points along the circumference, and the mean value was calculated. The average diameter and thickness were used to calculate the specimen volume (V).

Each group of samples was then separately immersed in 50 mL of distilled water in a marked incubator at 37 °C for 7 days. After the immersion period, the samples were removed, rinsed with water, gently blotted dry with absorbent paper to remove visible surface moisture, and weighed immediately. This saturated mass was recorded as m₂.

To calculate the final water sorption values, the samples were subjected to a second dehydration cycle (identical to the first). This final dry mass was recorded as m₃.

Water sorption (Wsp) was calculated using the following formula:

$$\mathrm{Wsp} = (\mathrm{m}_2 - \mathrm{m}_3)/\mathrm{V} $$

where m₂ is the mass of the specimen after water immersion, m₃ is the mass of the re-dried specimen, and V is the volume of the specimen.

### Color stability

Sample preparation followed the protocol described in the specimen preparation section.

Baseline color measurement: Before immersion, baseline color measurements were taken using a colorimeter (Nix Pro 2; Nix Sensor Ltd., Hamilton, Ontario, Canada) against a white background. The reliability and accuracy of this device for color measurement have been validated in previous studies [17]. The color values were recorded in the CIE L*a*b* system relative to standard illuminant A. In this system, L* refers to the lightness coordinate, which ranges in value from 0 (black) to 100 (white), and a* and b* are chromaticity coordinates on the green–red (− a* = green; +a* = red) and blue–yellow (− b* = blue; +b* = yellow) axes, respectively [18]. Measurements were repeated three times for each specimen at random points, and the mean values of L*, a*, and b* were calculated.

Immersion protocol: Specimens from each material (*n* = 10) were randomly divided into two subgroups (*n* = 5 per solution) and immersed in the following media:

- Coffee solution: Prepared by dissolving 2.5 g of Nescafé Taster’s Choice House Blend (Instant Coffee, Nestlé USA, Arlington, VA, USA) in 250 mL of boiled distilled water. The solution was stirred for 10 min at 250 rpm.
- Turmeric solution: Prepared by mixing 3.61 g of Turmeric powder (Pure ground spices, Gajanand Foods Pvt. Ltd., Gandhinagar, Gujarat, India) in 250 mL of boiled distilled water, stirred for 10 min at 250 rpm.

Each group specimen was stored in individual vials containing 50 mL of the respective solution in an incubator at 37 °C for a total duration of 28 days. To prevent precipitation and contamination, solutions were refreshed daily. Before placing samples into a fresh solution, they were rinsed with distilled water for 1 min.

#### Color assessment

Color measurements were performed at four time points: Baseline (0 days), 7 days, 14 days, 21 days, and 28 days of immersion. For the turmeric protocol, the 7-day immersion simulates approximately one year of dietary staining [19]. Consequently, the 28-day immersion period replicates approximately 4 years of exposure to turmeric-containing diets. And for the coffee protocol, the 7-day immersion simulates approximately 7–8 months of clinical exposure. Consequently, the 28-day immersion period replicates approximately 28–32 months (roughly 2.5 years) of exposure to coffee [20].

At each interval, the specimens were removed, rinsed, and dried, and post-staining values L*, a*, and b* of each sample were measured three times, from which the mean values were calculated. The total color change (ΔE) for each time point was calculated using the Euclidean distance formula:

$$\Delta\mathrm{E} = \sqrt{}[(\Delta\mathrm{L}^{*})^2 + (\Delta\mathrm{a}^{*})^2 + (\Delta\mathrm{b}^{*})^2]$$

Where ΔL*, Δa*, and Δb* are the differences between the baseline and post-immersion values at that specific time point. A ΔE value of < 3.3 was considered the threshold for clinically acceptable color change in this study [18].

### Statistical analysis

Statistical analysis was performed using SPSS software (version 26.0; IBM Corp., Armonk, NY, USA). Data were analyzed using one-way ANOVA followed by Tukey’s HSD post-hoc test to identify significant differences between groups. For color stability analysis, two-way ANOVA was employed to evaluate the effects of resin formulation (Group) and storage duration (Time), as well as their interaction (Group × Time). A significance level of *p* < 0.05 was set for all analyses. All experiments were performed with five replicates per group, and results are presented as mean ± standard deviation. A post-hoc power analysis conducted using G*Power 3.1.9.7 (one-way ANOVA, fixed effects) with an effect size of f = 1.60, α = 0.05, and a total sample size of *N* = 20 (4 groups, *n* = 5 per group) yielded a statistical power of 0.9999, confirming that the sample size was adequate to detect significant differences among groups.

## Results

### Contact angle and surface free energy

**Fig. 1:**
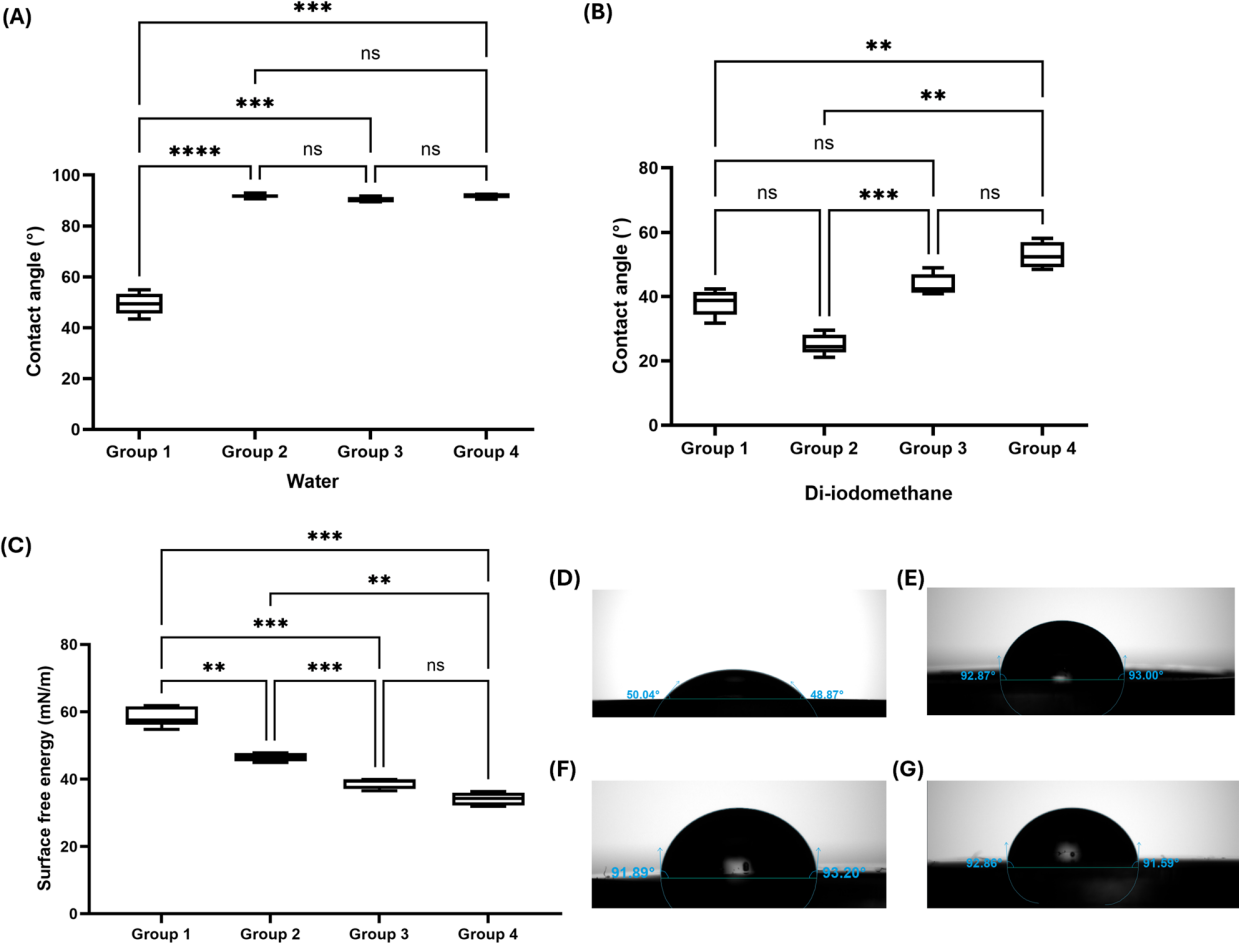
Contact angle and surface free energy measurements. (**A**) Water contact angle showing significantly higher values for all UDMA/IBOMA groups compared to the Bis-GMA/TEGDMA control (*p* < 0.0001, *p* < 0.001). (**B**) Di-iodomethane contact angle showing significant differences between groups (*p* < 0.01, *p* < 0.001). (**C**) Surface free energy demonstrating a progressive decrease with increasing IBOMA content; all experimental groups showed significantly lower SFE than the control (*p* < 0.01, *p* < 0.001). (**D**–**G**) Representative images of water droplet contact angles on specimen surfaces: (**D**) Group 1 showing a hydrophilic surface (~ 50°), (E) Group 2 (~ 93°), (**F**) Group 3 (~ 92°), and (**G**) Group 4 (~ 91°), all demonstrating hydrophobic surfaces (> 90°). ns = not significant

Water contact angle measurements revealed significant differences among the four groups (Fig. 1A). The control group (Group 1, Bis-GMA/TEGDMA) exhibited the lowest water contact angle at approximately 49°, indicating a hydrophilic surface. In contrast, all experimental UDMA/IBOMA groups demonstrated significantly higher water contact angles exceeding 90°, classifying them as hydrophobic surfaces (*p* < 0.0001). Groups 2, 3, and 4 showed water contact angles of approximately 95°, 95°, and 93°, respectively, with no significant differences among the experimental groups.

Di-iodomethane contact angle measurements (Fig. 1B) showed a different pattern. Group 3 exhibited significantly lower di-iodomethane contact angles compared to Groups 2 and 4 (*p* < 0.05), while Group 1 showed intermediate values.

Surface free energy (SFE) demonstrated a significant dependence on resin chemistry (Fig. 1C). The control group exhibited the highest SFE at 58.58 ± 2.97 mN/m. The experimental groups displayed a stepwise reduction in surface energy: Group 2 (46.53 ± 1.23 mN/m), Group 3 (38.68 ± 1.50 mN/m), and Group 4 (34.10 ± 1.90 mN/m). Tukey’s post-hoc analysis confirmed that all experimental groups possessed significantly lower SFE than the control (*p* < 0.01). Furthermore, significant differences were observed between Groups 2 and 3, and between Groups 2 and 4 (*p* < 0.05), while Groups 3 and 4 were not significantly different from each other. Representative images of water droplets on each material surface are shown in Figs. 1D–G.

### Degree of conversion

**Fig. 2:**
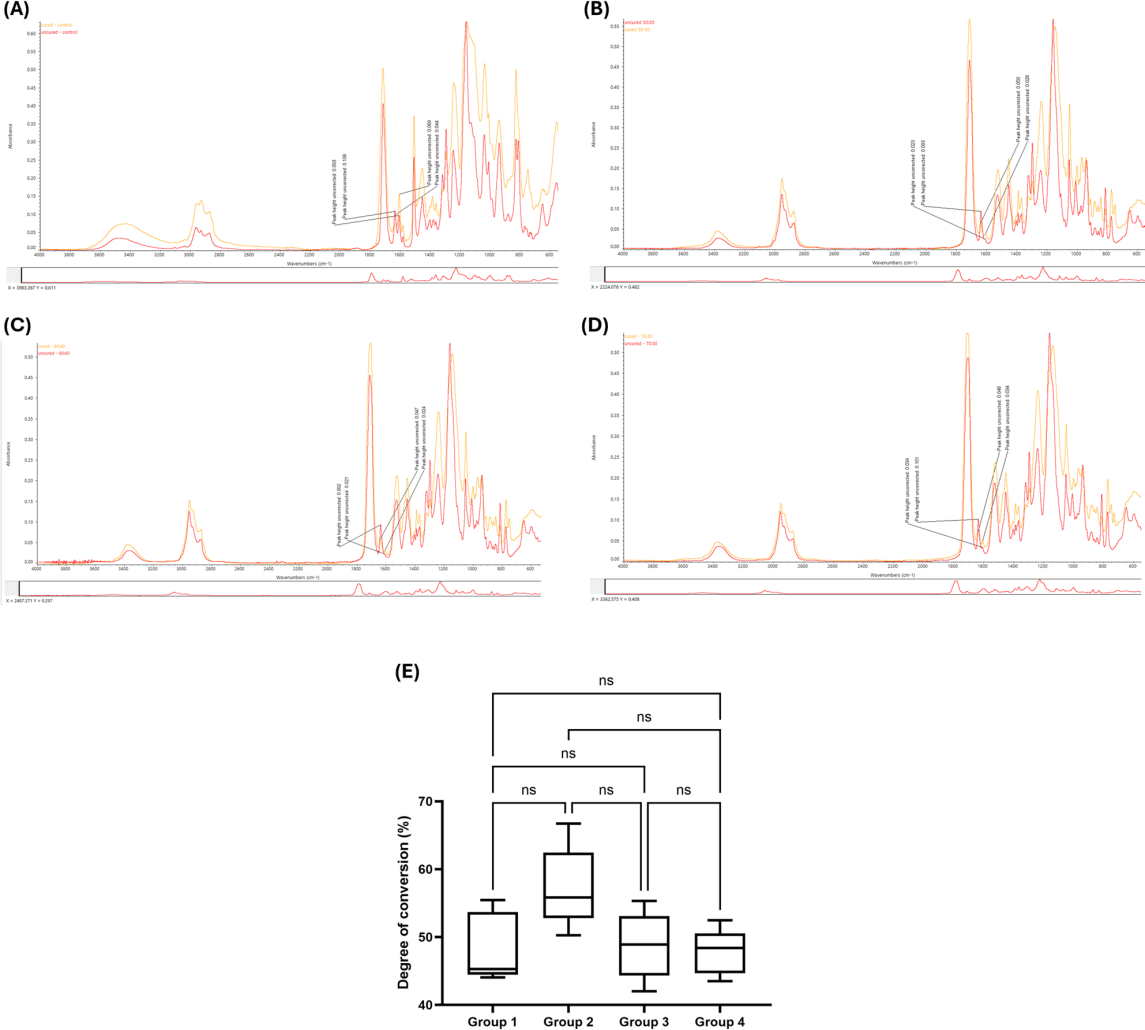
Degree of conversion analysis. (**A**–**D**) Representative FTIR spectra showing uncured (red) and cured (orange) specimens for (**A**) Group 1 (Bis-GMA/TEGDMA control), (**B**) Group 2 (UDMA/IBOMA 50:50), (**C**) Group 3 (UDMA/IBOMA 60:40), and (**D**) Group 4 (UDMA/IBOMA 70:30). The aliphatic C = C peak at 1637 cm⁻¹ and aromatic C–C reference peak at 1608 cm⁻¹ are indicated. (**E**) Degree of conversion calculated from FTIR spectra showing no statistically significant differences among groups. ns = not significant

FTIR spectra for uncured and cured specimens of each group are presented in Figs. 2A–D. The degree of conversion was calculated from the change in the aliphatic C = C peak relative to the aromatic C–C reference peak. As shown in Fig. 2E, no statistically significant differences in degree of conversion were observed among the four groups (*p* > 0.05). The control group (Group 1) showed a DC of approximately 50%, while Groups 2, 3, and 4 exhibited values of approximately 60%, 55%, and 55%, respectively. Although Group 2 (UDMA/IBOMA 50:50) showed the highest mean DC value, the differences did not reach statistical significance.

### Surface microhardness

**Fig. 3:**
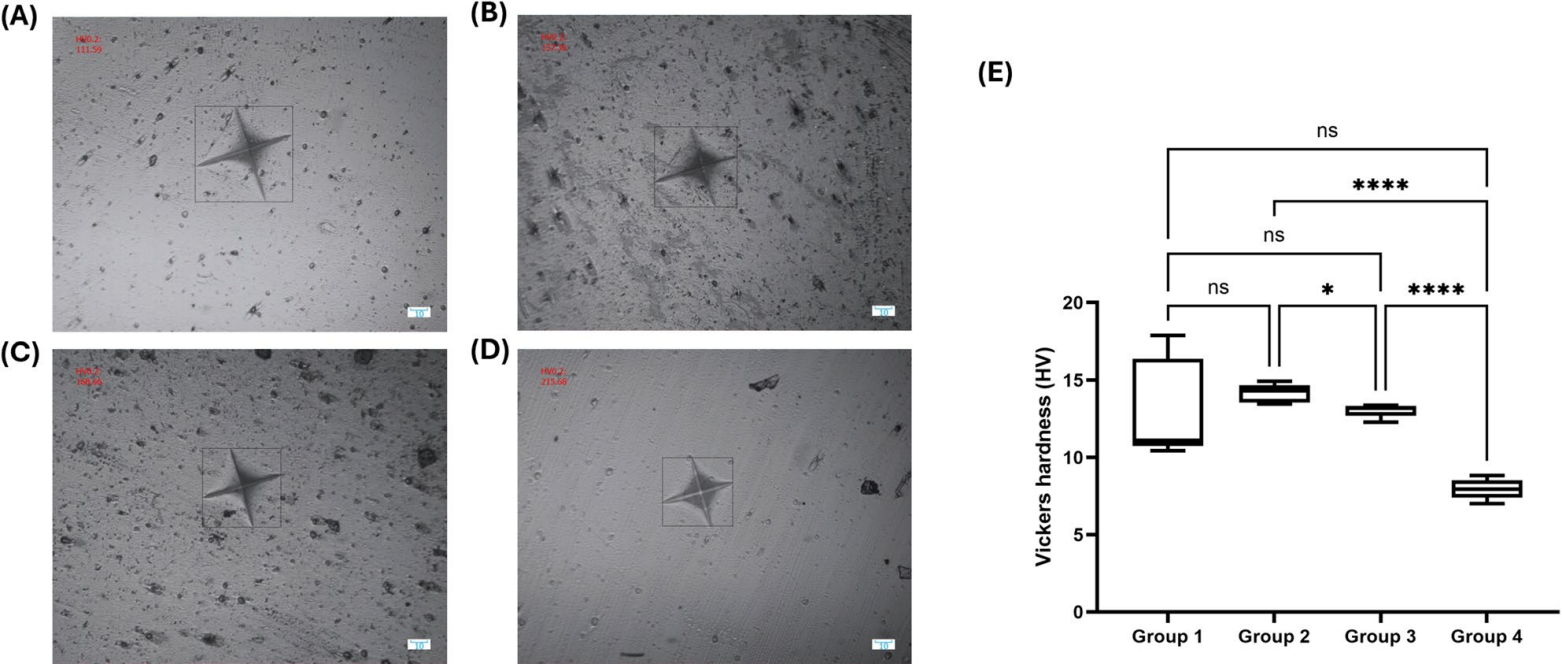
Surface microhardness analysis. (**A**–**D**) Representative light microscopy images of Vickers indentations on specimen surfaces: (**A**) Group 1, (**B**) Group 2, (**C**) Group 3, and (**D**) Group 4. (**E**) Surface microhardness values showing that Groups 1, 2, and 3 exhibited comparable hardness, while Group 4 demonstrated significantly lower hardness (*p* < 0.05, *p* < 0.0001). ns = not significant

Vickers microhardness varied significantly with resin composition (Fig. 3). Representative micrographs of Vickers indentations are shown in Figs. 3A–D. The control group (14.40 ± 5.14 HV), Group 2 (14.16 ± 0.60 HV), and Group 3 (13.05 ± 0.44 HV) exhibited comparable hardness values with no statistically significant differences among them. Group 4 demonstrated substantially lower hardness (7.96 ± 0.67 HV), differing significantly from the control (*p* < 0.0001), Group 2 (*p* < 0.0001), and Group 3 (*p* < 0.05) (Fig. 3E). These findings indicate that excessive IBOMA content (70:30 UDMA/IBOMA) compromises the mechanical integrity of the resin, whereas 50:50 and 60:40 formulations maintain hardness comparable to the conventional Bis-GMA/TEGDMA system.

### Water sorption

**Fig. 4:**
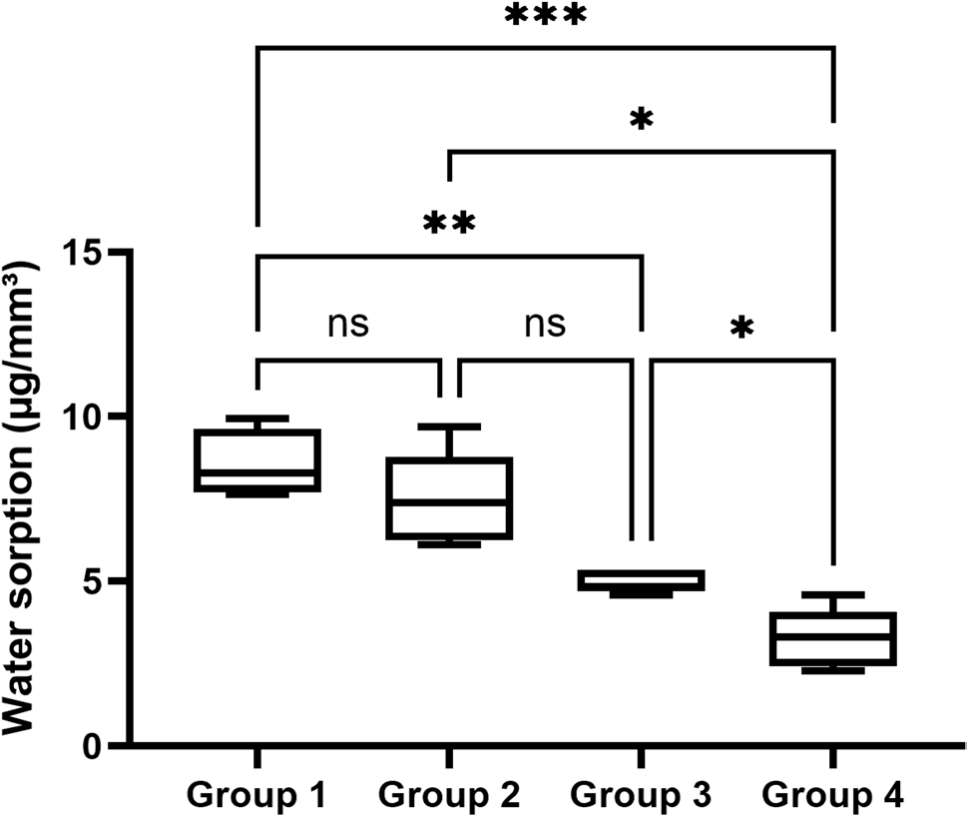
Water sorption values (µg/mm³) for the four experimental groups. Water sorption decreased progressively with increasing IBOMA content. Group 3, 4 showed a statistically significant reduction compared to the control (*p* < 0.01, *p* < 0.001). Compared with Group 2 and Group 3, Group 4 exhibited significantly lower water sorption (*p* < 0.05 for both comparisons). ns = not significant

Water sorption values are presented in Fig. 4. The control group demonstrated the highest sorption (11.26 ± 6.16 µg/mm³). Water sorption decreased progressively with increasing IBOMA content: 7.49 ± 1.42 µg/mm³ for Group 2, 4.99 ± 0.34 µg/mm³ for Group 3, and 3.26 ± 0.91 µg/mm³ for Group 4. Statistical analysis revealed that Groups 3 and 4 showed a significant reduction in water sorption compared to the control (*p* < 0.01, *p* < 0.001). Group 2 showed lower mean values compared to the control, but these differences did not reach statistical significance. A significant difference was also observed between Groups 3 & 4 and Groups 2 & 4(*p* < 0.05). These results indicate that higher IBOMA ratios effectively reduced water uptake compared to Bis-GMA/TEGDMA, with the 70:30 UDMA/IBOMA formulation showing the greatest improvement.

### Color stability

Color stability varied with immersion medium and resin formulation. Baseline CIE L*, a*, and b* values for all groups prior to immersion are summarized in Table 2. Notably, UDMA/IBOMA formulations demonstrated lower baseline b* values compared to the Bis-GMA/TEGDMA control, reflecting a more neutral baseline chromaticity that has implications for the interpretation of subsequent color change measurements.

**Table 2:** Baseline CIE L*, a*, and b* color parameter values of experimental resin specimens prior to staining solution immersion (mean ± SD, *n* = 5)

Group	Staining Solution	L*	a*	b*
Group 1	Coffee	68.9600 ± 2.1347	-2.8120 ± 0.1558	7.9160 ± 0.9276*
	Turmeric	67.2600 ± 3.9498	-2.6800 ± 0.3344	8.4300 ± 1.9120*
Group 2	Coffee	68.8320 ± 0.9987	-2.5420 ± 0.5970	5.3020 ± 2.8360
	Turmeric	70.8160 ± 0.6499	-2.5560 ± 0.3279	4.9940 ± 1.6889
Group 3	Coffee	69.6720 ± 2.5734	-2.5520 ± 0.2090	5.1140 ± 0.7826
	Turmeric	70.9660 ± 0.9119	-2.5020 ± 0.2625	4.9620 ± 1.1652
Group 4	Coffee	68.2260 ± 1.5639	-2.0620 ± 0.4188	5.8160 ± 1.4493
	Turmeric	68.5120 ± 1.0014	-1.4340 ± 1.7608	5.2840 ± 1.7146

Asterisk(*) denotes a statistically significant difference in b* values compared to experimental groups (*p* < 0.05)

#### Coffee immersion

**Fig. 5:**
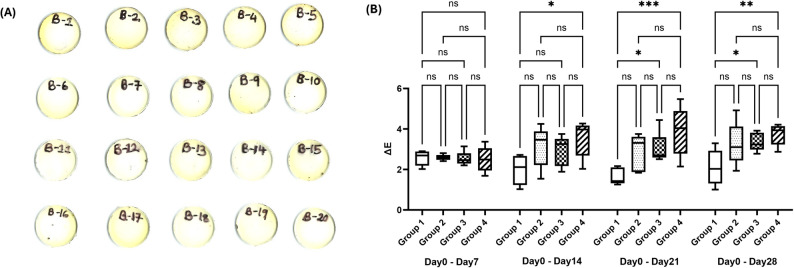
Color stability of resin composite specimens following coffee immersion. (**A**) Photographs of all specimens after 28-day coffee immersion (Group 1: B1–B5; Group 2: B6–B10; Group 3: B11–B15; Group 4: B16–B20). (**B**) Cumulative color change (ΔE) measured from baseline at each time point. *p* < 0.05, *p* < 0.01, *p* < 0.001; ns = not significant

Two-way ANOVA analysis of ΔE values revealed that significant differences in color stability were dependent on the duration of immersion, as shown in Fig. 5. During the initial Day 0–7 interval, all groups exhibited comparable color changes, with no statistically significant differences observed (*p* > 0.05). However, as the immersion period extended, the experimental formulations (Groups 2, 3, and 4) demonstrated significantly higher ΔE values compared to Group 1. This significant increase in color change relative to Group 1 was observed at the cumulative intervals of Day 0–14 (*p* < 0.05), Day 0–21(*p* < 0.001), and Day 0–28 (*p* < 0.01). Conversely, comparisons among the experimental groups themselves (Groups 2, 3, and 4) revealed no significant differences at any time point (*p* > 0.05), indicating that while they showed less color stability than Group 1, they performed equivalently to one another.

#### Turmeric immersion

**Fig. 6:**
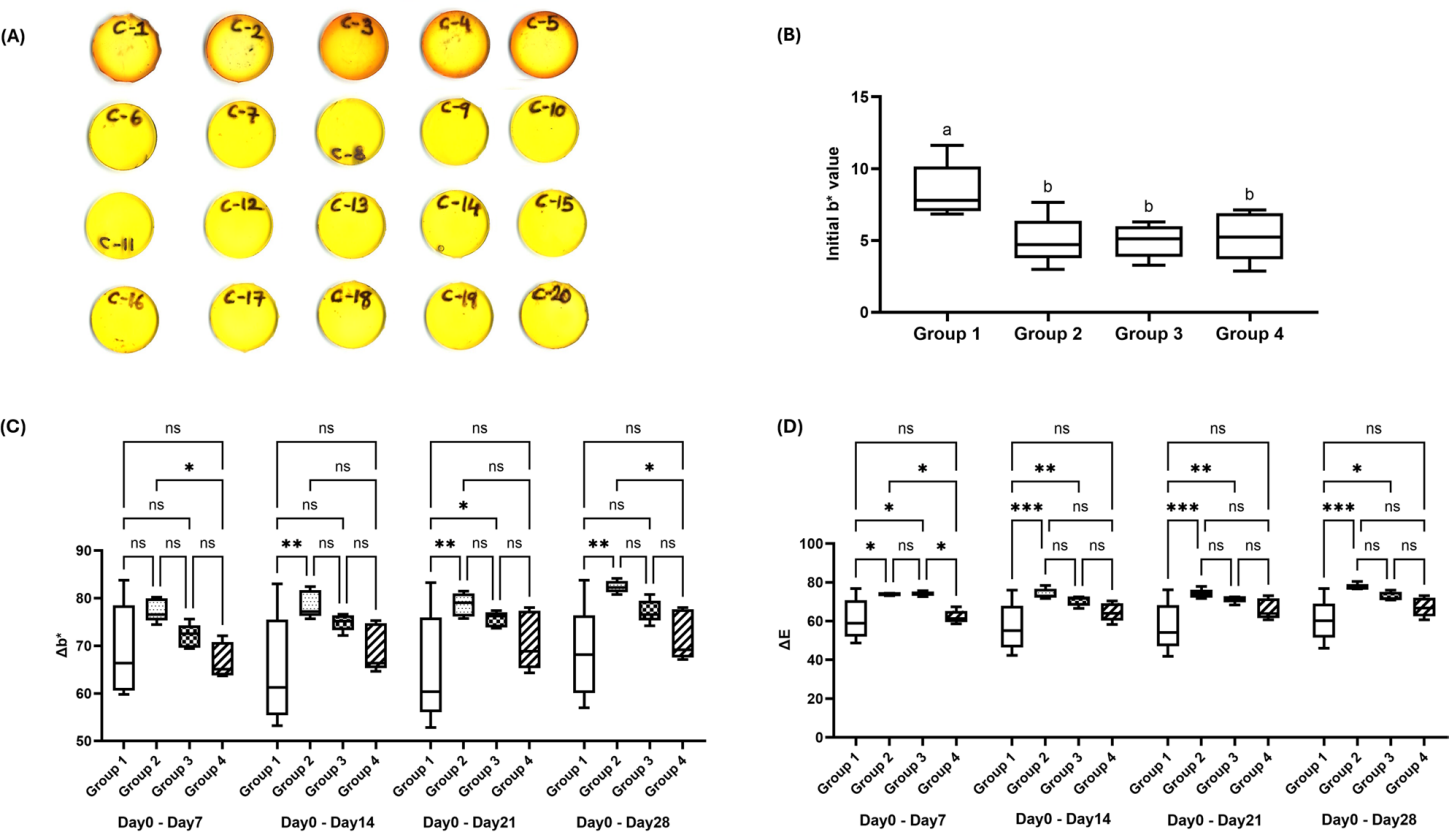
Color stability of resin composite specimens following turmeric immersion. (**A**) Photographs of all specimens after 28-day turmeric immersion (Group 1: C1–C5; Group 2: C6–C10; Group 3: C11–C15; Group 4: C16–C20). (**B**) Initial b* values (yellow-blue axis) before staining; different letters indicate statistically significant differences (*p* < 0.05). (**C**) Change in b* values (Δb*) from baseline over time. (**D**) Cumulative color change (ΔE) from baseline. *p* < 0.05, *p* < 0.01, *p* < 0.001; ns = not significant

Two-way ANOVA revealed significant main effects and interaction for resin formulation and storage duration on both ΔE and Δb^∗^ values (*p* < 0.0001), as shown in Fig. 6A-D. Regarding total color change (ΔE), significant differences were confined strictly to the initial immersion phase of Day 0–7, where Group 2 exhibited significantly higher values compared to Group 4 (*p* < 0.05). Beyond Day 7, cumulative color changes equilibrated, and no significant differences were detected for the cumulative intervals of Day 0–14, Day 0–21, and Day 0–28 (*p* > 0.05). In terms of chromatic shifts (Δb^∗^), significant differences extended through the Day 7–14 interval. All groups surpassed the ΔE > 3.3 clinical threshold, which is consistent with the known extreme chromogenic potency of curcumin and should be interpreted accordingly. Group 2 exhibited significantly higher Δb^∗^ values compared to Group 4 during the initial Day 0–7 phase (*p* < 0.0001), and this trend persisted into the Day 7–14 interval (*p* < 0.05). However, beyond Day 14, staining rates equilibrated, and no statistically significant differences were observed across subsequent time points (Day 14–21 and Day 21–28).

## Discussion

The clinical performance of dental restorative materials is fundamentally dependent on their ability to tolerate the chemically aggressive, moisture-rich, and thermally dynamic oral environment. While conventional composite development has historically prioritized optimization of the inorganic filler phase to enhance mechanical strength and wear resistance, accumulating evidence demonstrates that long-term clinical degradation is predominantly governed by the organic resin matrix [21]. The polymer network constitutes the phase most vulnerable to water sorption, hydrolytic scission, plasticization, and interfacial debonding at the filler-resin interface. Consequently, engineering the resin chemistry to minimize water interaction represents a more direct and effective strategy for improving restoration longevity than filler modification alone. Within this framework, the present study systematically evaluated how targeted modification of the resin phase using a UDMA/IBOMA formulation influences critical physicochemical properties governing hydrolytic stability, polymerization behavior, mechanical integrity, and chromatic resistance. Before interpreting these findings, however, it is necessary to acknowledge that the present investigation was performed on unfilled resin matrices, and the results should be understood within this context. In clinical composite systems, the inorganic filler phase, its loading fraction, particle characteristics, and the integrity of the filler-matrix interface collectively shape material behavior in ways that cannot be fully captured by resin-only testing.

The most pronounced outcome of this investigation was the substantial enhancement in surface hydrophobicity achieved across all UDMA/IBOMA formulations. The Bis-GMA/TEGDMA control exhibited a water contact angle of 49.45° ± 4.34°, characteristic of a hydrophilic surface, whereas all experimental groups demonstrated contact angles exceeding the hydrophobic threshold (> 90°), reaching 95.47° ± 2.45° in the 50:50 formulation. This transformation is directly attributable to IBOMA’s hydrocarbon-rich bicyclic structure lacking polar functional groups, contrasting sharply with TEGDMA’s multiple ether linkages that serve as hydrogen bond acceptors [22]. The concurrent reduction in surface free energy from 58.58 ± 2.97 mN/m in the control to 34.10 ± 1.90 mN/m in the 70:30 UDMA/IBOMA formulation further corroborates this molecular-level transformation. From a thermodynamic perspective, surfaces exhibiting lower surface free energy minimize the driving force for liquid spreading and adhesion, which has direct clinical implications for early bacterial colonization, biofilm maturation, and pigment retention [23, 24]. Hydrophobic surfaces create energetically unfavorable conditions for salivary protein adsorption and bacterial adhesion, potentially reducing secondary caries risk and marginal discoloration over extended clinical service.

The enhancement in surface hydrophobicity was accompanied by substantial reductions in bulk water sorption, underscoring the continuity between surface chemistry and three-dimensional network hydration behavior. The Bis-GMA/TEGDMA control absorbed 11.26 ± 6.16 µg/mm³ of water following seven-day immersion, whereas progressive incorporation of IBOMA reduced sorption in a dose-dependent manner, culminating in 3.26 ± 0.91 µg/mm³ for the 70:30 formulation, a 71% reduction relative to the control. These findings align with recent investigations by Pereira et al., who reported that substituting TEGDMA with IBOMA in flowable composite formulations significantly reduces water sorption. In their study, the hydrophobicity of the isobornyl ring was shown to effectively limit the diffusion of water molecules into the resin matrix, a phenomenon mirrored in our results where the 70:30 UDMA/IBOMA formulation (Group 4) demonstrated the greatest improvement in moisture resistance [25].

The clinical ramifications of reduced water sorption are profound. Water absorbed into the resin matrix acts as a plasticizer, disrupting van der Waals forces between polymer chains, reducing glass transition temperature, and compromising mechanical properties over time [26]. Furthermore, water-mediated hydrolysis of ester linkages and degradation of silane coupling agents at the filler-resin interface progressively undermine structural integrity and optical stability [27]. By substantially mitigating water uptake, UDMA/IBOMA formulations address a fundamental mechanism of long-term composite degradation.

Polymerization efficiency, quantified by degree of conversion, remained statistically comparable across all formulations, indicating that increased hydrophobicity did not compromise curing behavior. The control exhibited a DC of 48.31 ± 5.06%, while experimental groups ranged from 54.72 ± 8.50% to 57.26 ± 6.03%, with the 50:50 UDMA/IBOMA formulation showing the highest mean value. The conversion values observed in the present study fall within the lower range of those reported for experimental dimethacrylate systems, which may be partly attributed to the absence of a filler phase in the neat resin matrices, eliminating the internal light reflection contribution that can enhance polymerization efficiency in filled composite systems [28]. Although these differences did not achieve statistical significance, the numerical trend suggests that IBOMA’s monofunctional nature may delay network vitrification, maintaining a more fluid reaction environment that allows pendant radicals an extended time to react with unreacted double bonds [29]. Importantly, these results demonstrate that hydrophobicity enhancement through monomer substitution does not necessitate compromising polymerization completeness, a critical consideration for clinical translation, as incomplete conversion correlates with residual monomer elution, reduced mechanical properties, and cytotoxicity concerns [30]. However, given the variability observed in DC measurements, these non-significant differences should be interpreted cautiously. These results are particularly noteworthy when compared to recent investigations by Pereira et al., who observed that substituting TEGDMA with IBOMA in Bis-GMA-based flowable composites led to a reduction in DC. In contrast, our findings suggest that pairing IBOMA with a UDMA base may mitigate this typical trade-off [25].

Mechanical evaluation via Vickers microhardness revealed an anticipated structure-property relationship between monomer functionality and network rigidity. The control (14.40 ± 5.14 HV), 50:50 formulation (14.16 ± 0.60 HV), and 60:40 formulation (13.05 ± 0.44 HV) exhibited statistically equivalent hardness, whereas the 70:30 UDMA/IBOMA system demonstrated significantly reduced values (7.96 ± 0.67 HV). This decline reflects IBOMA’s monofunctional architecture limiting network crosslinking capacity. As IBOMA concentration increases, effective crosslink density decreases, resulting in longer, more flexible polymer chains with fewer covalent interconnections [31]. However, surface microhardness represents only one dimension of mechanical competence and correlates imperfectly with clinically relevant properties such as fracture toughness, fatigue resistance, and marginal adaptation under cyclic loading [32]. For restorative applications where hydrolytic stability and interfacial integrity supersede absolute stiffness—such as cervical lesions, Class V restorations, or liners beneath indirect restorations, the benefits conferred by enhanced hydrophobicity may substantially outweigh modest reductions in hardness. The 50:50 and 60:40 formulations are particularly noteworthy in this regard, as they maintained surface hardness values statistically indistinguishable from the conventional control while yielding meaningful improvements in water resistance, suggesting these compositions may offer a more favorable property balance among the formulations investigated.

Color stability assessment revealed divergent performance patterns contingent upon the chromogenic challenge. Following coffee immersion, UDMA/IBOMA formulations exhibited significantly higher ΔE values than the Bis-GMA/TEGDMA control at the 14, 21, and 28-day intervals, with values exceeding the clinically acceptable threshold of 3.3 in several experimental groups. Coffee staining is mediated by the adsorption of amphiphilic polyphenolic compounds and penetration through surface irregularities [33]. The higher ΔE values observed in UDMA/IBOMA groups should not be attributed solely to greater chromogen uptake; rather, they reflect a combination of contributing factors. The lighter baseline color of these formulations (lower initial b*) creates a greater optical contrast against which chromogen uptake is perceived, amplifying the apparent color change relative to the inherently yellower Bis-GMA/TEGDMA control. Beyond this optical contrast effect, the more open network architecture associated with increasing IBOMA content and reduced crosslink density may further contribute to greater network permeability, facilitating chromophore infiltration independently of water uptake differences. In contrast, turmeric immersion produced pronounced discoloration across all formulations. UDMA/IBOMA blends exhibited a more neutral baseline color (lower initial b*), offering advantages for shade matching; however, this lighter baseline further amplified the apparent yellowing (Δb*) relative to the Bis-GMA/TEGDMA control through the same optical contrast mechanism described above. Within the experimental formulations, color stability improved with increasing UDMA content, with the 70:30 UDMA/IBOMA formulation exhibiting the lowest staining. This suggests that UDMA’s capacity for intermolecular hydrogen bonding contributes to a tighter polymer network that resists pigment penetration, whereas higher proportions of monofunctional diluents reduce crosslink density and compromise resistance to chromogen diffusion [34]. During the initial 0–7 day interval, Groups 2 and 3 exhibited significantly elevated ΔE values compared to the control and Group 4; however, beyond Day 7, color changes equilibrated across all groups. It should be acknowledged that turmeric represents an extreme and accelerated chromogenic challenge that likely overestimates staining encountered under typical intraoral conditions, and direct clinical extrapolation from these findings is therefore limited. These data are more appropriately interpreted as a comparative stress test of network resistance to pigment infiltration, underscoring the need for future staining models that incorporate cyclic dietary exposure, aging, and shaded resin matrices to better approximate long-term intraoral color behavior.

Collectively, these findings identify the 70:30 UDMA/IBOMA formulation as the most promising candidate evaluated in this study. Although this formulation exhibited reduced surface hardness, it demonstrated the lowest surface free energy (34.10 ± 1.90 mN/m) and greatest resistance to water sorption (3.26 ± 0.91 µg/mm³), parameters strongly associated with long-term clinical durability. For restorative applications prioritizing hydrolytic stability, marginal integrity, and esthetic longevity, such as cervical restorations, liners, or low-load-bearing posterior restorations, the benefits of enhanced hydrophobicity may outweigh modest reductions in surface hardness. Importantly, this formulation maintains acceptable polymerization behavior and remains free of Bis-GMA-related concerns, supporting its relevance for contemporary restorative dentistry [35].

Several limitations of this study should be acknowledged. The primary limitation is that this investigation was conducted exclusively on unfilled, neat resin matrices. Because dental composites are inherently biphasic materials, their clinical behavior is dictated by the complex synergy between the organic phase and the inorganic filler particles. Consequently, the findings reported here represent a fundamental assessment at the resin-matrix level and do not account for the critical influences of filler loading, particle morphology, or the hydrolytic stability of the silane coupling interface, all of which are primary drivers of composite performance and mechanical durability in the oral cavity. Second, the sample size of *n* = 5 per group was adopted in accordance with comparable resin characterization studies in the dental materials literature, and a post-hoc power analysis confirmed adequate statistical power for the comparisons performed (G*Power 3.1.9.7; f = 1.60, α = 0.05, power = 0.9999). Nevertheless, the possibility of Type II error cannot be fully excluded for non-significant comparisons, and such findings should be interpreted with caution rather than construed as evidence of true equivalence. Additionally, degree of conversion was assessed using ATR-FTIR based on the 1637/1608 cm⁻¹ band ratio, with baseline correction applied to all spectra prior to analysis. Nevertheless, overlapping spectral contributions from UDMA’s urethane-associated vibrations and potential ATR-related artifacts cannot be fully excluded, and future studies should consider cross-validation with Raman spectroscopy or real-time photorheometry for more comprehensive characterization of polymerization kinetics. Furthermore, accelerated aging and staining protocols may not fully replicate the complexity of intraoral conditions. Long-term thermocycling, mechanical fatigue, and biofilm-mediated degradation were not evaluated. Future studies should incorporate filled composite systems and advanced aging models that more closely simulate real-life clinical conditions, including thermal cycling, cyclic loading, and prolonged exposure to fluctuating oral environments, to better predict clinical behavior.

In conclusion, this study demonstrates that targeted modification of resin matrix chemistry represents a powerful and underutilized strategy for enhancing composite performance. By increasing matrix hydrophobicity through controlled UDMA/IBOMA formulations, substantial improvements in hydrolytic resistance and surface stability were achieved without compromising polymerization efficiency. The 70:30 UDMA/IBOMA system offers a compelling balance of properties and highlights the potential of resin-focused design for the development of next-generation, durable, BPA-free restorative materials. These findings provide a strong foundation for future translational research aimed at advancing composite longevity under clinically relevant conditions.

## Conclusions

This study establishes UDMA/IBOMA formulations as viable alternatives to conventional Bis-GMA/TEGDMA systems, offering a pathway toward more durable and biocompatible dental restorative materials. The experimental resins achieved true hydrophobic surfaces (contact angles > 90°) and demonstrated up to 71% reduction in water sorption compared to controls—a critical advancement given that water-mediated degradation is a major contributor to long-term composite failure. The 50:50 and 60:40 UDMA/IBOMA formulations demonstrated a more balanced overall property profile, combining meaningful improvements in water sorption and hydrolytic resistance with comparatively better surface microhardness and color stability, making them more suitable candidates for broader restorative applications. The 70:30 UDMA/IBOMA formulation achieved the greatest hydrolytic resistance and lowest water sorption; however, the associated reduction in surface microhardness and relatively inferior color stability in coffee immersion suggest that this formulation may be better suited for specific low-load clinical scenarios such as cavity liners, bases, or cervical restorations. Importantly, these BPA-free systems address growing concerns about biocompatibility without compromising polymerization efficiency or essential material properties. Translation of these laboratory findings to filled composite systems under simulated clinical conditions represents the logical next step toward delivering restorative materials capable of withstanding the demanding oral environment for extended service lifetimes.

## Supplementary Information

Supplementary Material 1.

## Data Availability

The datasets used and/or analyzed during the current study are available from the corresponding author on reasonable request.
