# Supplementary material for: Hydrophobic monomer systems for dental composites: development and physicochemical evaluation of UDMA/IBOMA formulations
Source: BMC Oral Health. 2026 Mar 14;26:718. doi: 10.1186/s12903-026-08088-x (PMC13101269; doi:10.1186/s12903-026-08088-x)
Supplement: Supplementary file 1 — Supplementary Material 1. [file 12903_2026_8088_MOESM1_ESM.docx]

**Table I:** ΔL*, Δa*, Δb*, and ΔE* values following coffee immersion at each time interval (mean ± SD, n = 5).

| **Group** | **Time interval** | ΔL* | Δa* | Δb* | ΔE* |
| --- | --- | --- | --- | --- | --- |
| Group 1 | T=7 days | -1.5740 ± 1.1815 | 0.1380 ± 0.2024 | 1.3780 ± 1.2004 | 2.5662 ± 0.3747 |
|  | T=14 days | -1.8140 ± 0.3582 | 0.0920 ± 0.0672 | 1.7980 ± 0.3682 | 2.5943 ± 0.1431 |
|  | T=21 days | -1.7760 ± 0.6336 | 0.2620 ± 0.0847 | 1.7040 ± 0.2660 | 2.5314 ± 0.3569 |
|  | T=28 days | -1.7720 ± 0.7223 | 0.6820 ± 0.4041 | 1.5100 ± 0.3752 | 2.4977 ± 0.6201 |
| Group 2 | T=7 days | -1.4920 ± 0.7015 | 0.0880 ± 0.1702 | 1.0440 ± 0.8871 | 1.9837 ± 0.7376 |
|  | T=14 days | -2.2260 ± 0.8422 | 0.0340 ± 0.3400 | 1.9900 ± 1.1366 | 3.1295 ± 1.0099 |
|  | T=21 days | -1.9600 ± 1.1719 | 0.3620 ± 0.1064 | 1.8340 ± 0.8424 | 2.9271 ± 0.7447 |
|  | T=28 days | -2.3300 ± 1.0520 | 0.7300 ± 0.3941 | 2.4680 ± 0.3619 | 3.5363 ± 0.9094 |
| Group 3 | T=7 days | -1.0160 ± 0.6588 | 0.4040 ± 0.1195 | 0.4060 ± 1.2060 | 1.6566 ± 0.4155 |
|  | T=14 days | -1.9400 ± 0.8613 | 0.2820 ± 0.2924 | 1.7200 ± 1.3101 | 2.8577 ± 0.9151 |
|  | T=21 days | -2.2300 ± 1.2408 | 0.5220 ± 0.0853 | 1.7160 ± 0.4657 | 3.0141 ± 0.8032 |
|  | T=28 days | -2.7580 ± 1.4005 | 0.8300 ± 0.3415 | 2.4120 ± 0.7435 | 3.8758 ± 1.2228 |
| Group 4 | T=7 days | -1.9740 ± 0.8138 | 0.3920 ± 0.1139 | 0.0500 ± 0.7339 | 2.0993 ± 0.8782 |
|  | T=14 days | -2.3580 ± 0.6126 | 0.3380 ± 0.3100 | 2.0420 ± 1.2553 | 3.2483 ± 1.0786 |
|  | T=21 days | -2.5540 ± 0.6671 | 0.2760 ± 0.3355 | 2.0740 ± 0.4307 | 3.3664 ± 0.4510 |
|  | T=28 days | -2.9020 ± 0.5907 | 0.8800 ± 0.3443 | 2.1400 ± 0.2367 | 3.7369 ± 0.5366 |

**Table II:**ΔL*, Δa*, Δb*, and ΔE* values following turmeric immersion at each time interval (mean ± SD, n = 5).

| **Group** | **Time interval** | ΔL* | Δa* | Δb* | ΔE* |
| --- | --- | --- | --- | --- | --- |
| Group 1 | T=7 days | -3.5660 ± 0.6152 | -5.7540 ± 1.2918 | 60.4680 ± 10.7930 | 60.8795 ± 10.6492 |
|  | T=14 days | -2.7960 ± 1.0084 | -8.2100 ± 0.6865 | 72.4320 ± 2.8167 | 73.8910 ± 0.5663 |
|  | T=21 days | -3.8160 ± 1.1544 | -8.8840 ± 0.5860 | 67.1020 ± 3.6755 | 74.1099 ± 1.1967 |
|  | T=28 days | -4.5580 ± 0.6663 | -7.1340 ± 1.0797 | 61.5480 ± 3.4545 | 62.1417 ± 3.3659 |
| Group 2 | T=7 days | -3.7860 ± 1.7950 | -6.1580 ± 2.2573 | 56.1760 ± 12.7481 | 56.7353 ± 12.5387 |
|  | T=14 days | -3.7540 ± 0.4214 | -7.6480 ± 1.1890 | 73.6200 ± 2.7736 | 74.1213 ± 2.7276 |
|  | T=21 days | -4.1580 ± 1.2589 | -8.2520 ± 0.6701 | 69.9480 ± 2.6350 | 70.5697 ± 2.5499 |
|  | T=28 days | -4.0980 ± 0.9541 | -7.7400 ± 1.1736 | 64.0080 ± 4.9301 | 64.6248 ± 4.8269 |
| Group 3 | T=7 days | -3.2400 ± 1.0910 | -5.8340 ± 2.8081 | 56.4480 ± 12.8708 | 56.9355 ± 12.7005 |
|  | T=14 days | -3.9400 ± 0.8134 | -7.1120 ± 1.4708 | 73.6640 ± 2.4576 | 74.1273 ± 2.4285 |
|  | T=21 days | -3.8760 ± 1.1072 | -8.1660 ± 0.5977 | 70.6480 ± 1.7142 | 71.2333 ± 1.6860 |
|  | T=28 days | -4.1100 ± 1.8300 | -7.3300 ± 1.5923 | 65.5160 ± 5.5636 | 66.0984 ± 5.4123 |
| Group 4 | T=7 days | -3.7600 ± 1.4807 | -5.5780 ± 2.6948 | 59.7820 ± 11.2286 | 60.2441 ± 11.0803 |
|  | T=14 days | -3.8740 ± 0.8197 | -5.9600 ± 1.5554 | 77.4140 ± 1.5487 | 77.7547 ± 1.6026 |
|  | T=21 days | -4.0920 ± 1.4226 | -7.7400 ± 0.8701 | 72.2540 ± 2.3505 | 72.8004 ± 2.2471 |
|  | T=28 days | -4.5540 ± 1.1315 | -7.0900 ± 1.3469 | 66.6220 ± 5.1795 | 67.1777 ± 5.0730 |
